# Evidence that phosphatidylinositol 3-kinase is involved in sperm-induced tyrosine kinase signaling in *Xenopus *egg fertilization

**DOI:** 10.1186/1471-213X-9-68

**Published:** 2009-12-17

**Authors:** Gunay Mammadova, Tetsushi Iwasaki, Alexander A Tokmakov, Yasuo Fukami, Ken-ichi Sato

**Affiliations:** 1 The Graduate School of Science, Kobe University, 1-1 Rokkodai-cho, Nada-ku, Kobe 657-8501, Japan; 2 Department of Biotechnology, Faculty of Engineering, Kyoto Sangyo University, Kamigamo-Motoyama, Kita-ku, Kyoto 603-8555, Japan; 3 Research Center for Environmental Genomics, Kobe University, 1-1 Rokkodai-cho, Nada-ku, Kobe 657-8501, Japan

## Abstract

**Background:**

Studies have examined the function of PI 3-kinase in the early developmental processes that operate in oocytes or early embryos of various species. However, the roles of egg-associated PI 3-kinase and Akt, especially in signal transduction at fertilization, are not well understood.

**Results:**

Here we show that in *Xenopus *eggs, a potent inhibitor of phosphatidylinositol 3-kinase (PI 3-kinase), LY294002 inhibits sperm-induced activation of the tyrosine kinase Src and a transient increase in the intracellular concentration of Ca^2+ ^at fertilization. LY294002 also inhibits sperm-induced dephosphorylation of mitogen-activated protein kinase, breakdown of cyclin B2 and Mos, and first embryonic cleavage, all of which are events of Ca^2+^-dependent egg activation. In fertilized eggs, an 85-kDa subunit of PI 3-kinase (p85) undergoes a transient translocation to the low-density, detergent-insoluble membranes (membrane microdomains) where Src tyrosine kinase signaling is operating. However, the tyrosine phosphorylation of p85 in fertilized eggs is not as evident as that in H2O2-activated eggs, arguing against the possibility that PI 3-kinase is activated by Src phosphorylation. Nevertheless, sperm-induced activation of PI 3-kinase has been demonstrated by the finding that Akt, a serine/threonine-specific protein kinase, is phosphorylated at threonine-308. The threonine-phosphorylated Akt also localizes to the membrane microdomains of fertilized eggs. Application of bp(V), an inhibitor of PTEN that dephosphorylates PIP3, the enzymatic product of PI 3-kinase, promotes parthenogenetic activation of *Xenopus *eggs. In vitro kinase assays demonstrate that PIP3 activates Src in a dose-dependent manner.

**Conclusions:**

These results suggest that PI 3-kinase is involved in sperm-induced egg activation via production of PIP3 that would act as a positive regulator of the Src signaling pathway in *Xenopus *fertilization.

## Background

At fertilization, the union of egg and sperm promotes a series of biochemical and cell biological changes within the fertilized egg. This phenomenon is termed 'egg activation' [1-3]. A trigger of egg activation, which acts inside the fertilized egg after the egg-sperm union, is a transient increase in intracellular Ca^2+ ^(Ca^2+ ^transient) [4-6]. One important consequence of egg activation is that the egg acquires the ability to exclude additional fertilizing sperm (block to polyspermy). In many, but not all species, the block to polyspermy is achieved by an altered membrane potential and/or by the formation of a fertilization envelope. Another important consequence is that the activated egg resumes meiotic cell division. In the case of amphibian and most mammalian species, the meiotic cell cycle of unfertilized eggs pauses at metaphase II, and successful fertilization promotes meiotic resumption and extrusion of the second polar body. These egg activation events are followed by the fusion of maternal and paternal nuclei and the initiation of embryonic cell division that produce an offspring.

The sperm-induced Ca^2+ ^transient, a key event in the initiation of egg activation, is commonly mediated by inositol 1,4,5-trisphosphate (IP3), a second messenger that is produced by the phospholipase C (PLC)-catalyzed hydrolysis of phosphatidylinositol 4,5-bisphosphate. The molecular mechanism operating between egg-sperm membrane interaction/fusion and the activation of PLC, however, varies among species: in mammals and the newt *Cynops pyrrohogaster*, introduction of the sperm-derived proteins PLCζ [7] and citrate synthase [8], respectively, may account for this task. In these cases, egg-sperm membrane fusion, rather than egg-sperm membrane interaction, is crucial for initiating the Ca^2+ ^transient. On the other hand, for some sea invertebrates, fish and frogs, there is still a debate over the mechanism by which the egg undergoes a Ca^2+ ^transient. That sequential activation of the egg-associated Src tyrosine kinase and PLCγ is required for the Ca^2+ ^transient in the sea urchin, starfish, fish, and frog [9-14] suggests that these species employ the membrane interaction machinery. Also, some membrane-associated molecules have been postulated as sperm-interacting and signal-transducing elements in *Xenopus *eggs [15-18].

Several studies have evaluated the function of PI 3-kinase in the early developmental processes that operate in oocytes or early embryos of various species. In *Xenopus*, PI 3-kinase and Akt are required for insulin-induced, but not progesterone-induced, oocyte maturation [19,20], although one report has shown a requirement of PI 3-kinase for progesterone-induced oocyte maturation [21]. There are also reports that the activation of γ-subspecies of PI 3-kinase [22] or application of wortmannin [23] induces oocyte maturation. On the other hand, oocyte maturation in the ascidian [24], mouse [25,26] and starfish [27] has been shown to require activity of PI 3-kinase. Oocyte-specific deletion of PTEN is shown to cause premature activation of the primordial follicle cells [28], suggesting that a precise level of PIP3 is important for this process.

Moreover, the importance of PI 3-kinase and/or Akt has been demonstrated in FGF-dependent signal transduction [29,30] and glucose transport in *Xenopus *oocytes [31], the first mitotic cell division in the sea urchin [32] and starfish [33], autocrine-mediated survival signaling of mouse two-cell embryos [34], mesoderm induction [35], gastrulation [36,37] and neurogenesis [38] in *Xenopus*. Collectively, these studies demonstrate the general importance of PI 3-kinase and its enzymatic products in several aspects of development. However, a study on egg-associated PI 3-kinase and Akt with a focus on fertilization signaling has yet to be done, though Mehlmann et al. [39] found that LY294002 does not inhibit Ca^2+ ^transients in fertilized mouse eggs.

Here, we provide evidence that the sperm-induced Ca^2+ ^transient requires the activity of the egg-associated PI 3-kinase in *Xenopus*. Many somatic cell systems utilize PI 3-kinase as a downstream effector of tyrosine kinase signaling [40,41]. In fertilized *Xenopus *eggs, however, tyrosine phosphorylation of the p85 subunit of PI 3-kinase is not evident. On the other hand, PI 3-kinase seems to act as an upstream, positive regulator for the Src-PLCγ pathway, because the PI 3-kinase inhibitor LY294002 inhibits the sperm-induced activation of Src, Ca^2+ ^transient, and subsequent egg activation events. Subcellular fractionation studies demonstrate that the 85-kDa subunit of PI 3-kinase undergoes a transient translocation to low-density, detergent-insoluble membranes (membrane microdomains), where Src is present even before fertilization, awaiting a fertilization signal from sperm [42]. Activation of PI 3-kinase in fertilized *Xenopus *eggs is suggested by the finding that Akt becomes phosphorylated at threonine-308 in fertilized eggs. These results highlight for the first time that activity of the egg PI 3-kinase may play an important role in signal transduction for fertilization.

## Results

**Figure 1:**
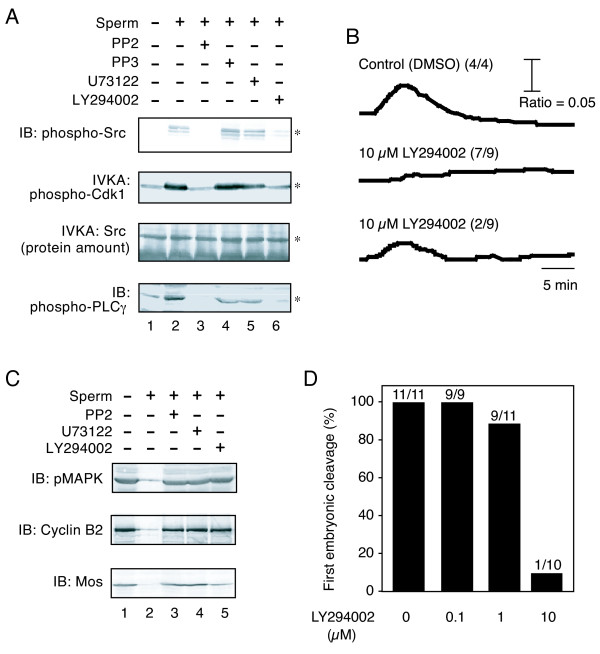
**Inhibition of early events of *Xenopus *egg fertilization by LY294002, a potent PI 3-kinase inhibitor**. (A) *Xenopus *unfertilized eggs were microinjected with DMSO alone (lanes 1 and 2), 10 μM PP2 (lane 3), 10 μM PP3 (lane 4), 10 μM U73122 (lane 5), or 10 μM LY294002 (lane 6), and subjected to no treatment (lane 1, - sperm) or insemination for 5 min (lanes 2-6, + sperm). SDS (0.1%)-solubilized egg membrane fractions (500 μg/lane) abundant in Src and PLCγ were analyzed for tyrosine phosphorylation of Src (top panel, IB: phospho-Src), activation of Src (middle panels, IVKA: phospho-Cdk1; IVKA: protein amounts of the immunoprecipitated Src), and tyrosine phosphorylation of PLCγ (bottom panel, IB: phospho-PLCγ) as described in "Methods". Asterisks in each panel indicate the positions of the protein bands of interest. (B) Shown are representative traces of sperm-induced Ca^2+ ^release, as monitored by the fluorescent ratio signal, in *Xenopus *eggs that were co-injected with fura-2 and LY294002 (0 or 10 μM) and inseminated as described in "Methods". (C) Triton X-100-solubilized extracts (100 μg/lane) were prepared from *Xenopus *eggs that were injected with the indicated inhibitors (lanes 3-5, each 10 μM) and inseminated for 40 min. Extracts from DMSO (0.2%)-injected unfertilized (lane 1) or fertilized eggs (lane 2) were also prepared as controls. Samples were separated by SDS-PAGE and analyzed by immunoblotting with antibodies against phospho-MAPK (top panel), cyclin B2 (middle panel), or Mos (bottom panel) as described in "Methods". (D) The occurrence of first embryonic cleavage was evaluated in fertilized *Xenopus *embryos that were injected with the indicated concentrations of LY294002 and inseminated for 100 min. Values indicated on each bar are the number of cleaved embryos per number of embryos tested.

We first examined the effect of LY294002, a potent PI 3-kinase inhibitor, on sperm-induced activation of the Src-PLCγ pathway. We microinjected LY294002 into unfertilized *Xenopus *eggs at a final concentration of 10 μM. The injected eggs were inseminated for 5 min, fractionated, and examined for sperm-induced activation and phosphorylation of Src and PLCγ, using immunoprecipitation, immunoblotting, and/or in vitro kinase assays (IVKA). LY294002 inhibited the sperm-induced activation of Src, as judged by the phosphorylation of tyrosine-419 in Src (Figure 1A, IB: phospho-Src, lanes 1, 2, and 6) and by the kinase activity toward a synthetic peptide substrate (Figure 1A, IVKA: phospho-Cdk1). Tyrosine phosphorylation of PLCγ was also inhibited by LY294002 (Figure 1A, IB: phospho-PLCγ). As we reported earlier, a Src-specific inhibitor PP2 also had an inhibitory effect (Figure 1A, lane 3). Neither the inactive analog PP3 (lane 4) nor U73122 (lane 5), a PLC-specific inhibitor, showed such an effect.

We have previously shown that activation of Src and PLCγ is necessary for sperm-induced activation of *Xenopus *eggs. Therefore, we examined the effect of LY294002 on the downstream events of the Src-PLCγ pathway; namely the transient Ca^2+ ^release (Figure 1B), inactivation of components of the cytostatic factor (Figure 1C), and first cell cleavage (Figure 1D). All of the LY294002-injected eggs exhibited features of an impaired transient Ca^2+ ^release, as monitored using the fluorescent signal of fura-2 that was introduced into the eggs (Figure 1B). Seven of nine eggs tested showed no transient Ca^2+ ^release, and two of nine showed a transient Ca^2+ ^release of decreased amplitude (Figure 1B). LY294002 also had an inhibitory effect on the Ca^2+^-dependent breakdown of the Mos protein kinase and cyclin B2 as well as dephosphorylation of mitogen-activated protein kinase (MAPK) (Figure 1C). These Ca^2+^-dependent events are required for cancelling the arrest of the second meiotic cell cycle and for starting embryonic cell division. Actually, eggs injected with 10 μM LY294002 failed to undergo normal first cell cleavage (Figure 1D).

We also examined the effect of LY294002 on egg activation, as evaluated by the occurrence of cortical contraction, induced by several artificial egg activators. LY294002 effectively inhibited the egg activation induced by cathepsin B at 5 U/ml (23 of 90 eggs showed cortical contraction). On the other hand, the egg activation induced by A23187 at 2 μM and that by H2O2 at 10 mM were not inhibited; 85 of 90 eggs and 76 of 90 eggs were activated, respectively. These data suggest that egg activation by cathepsin B, which mimics the action of sperm protease and requires the egg Src activity, also requires the activity of PI 3-kinase. It should be noted that we did not employ LY294002 higher than 10 μM, because it required more volume of DMSO solution to be injected that would show a toxic effect on eggs.

**Figure 2:**
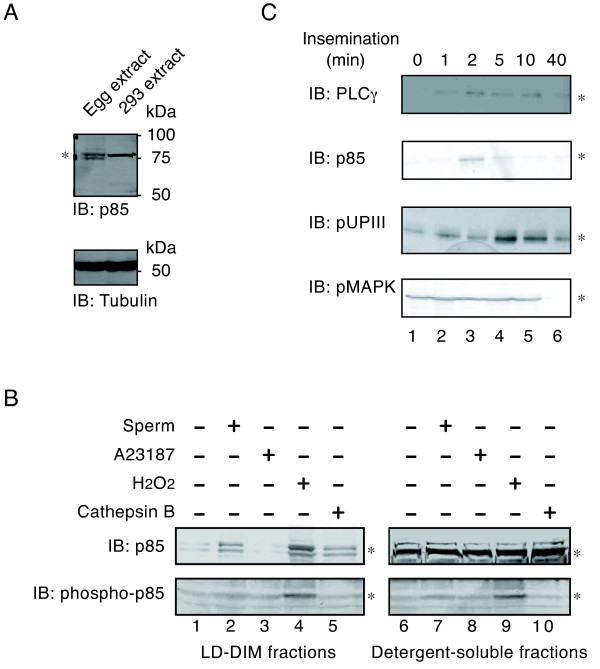
**Subcellular localization and tyrosine phosphorylation of an 85-kDa subunit of PI 3-kinase before and after fertilization of *Xenopus *eggs**. (A) Triton X-100-solubilized extracts of *Xenopus *unfertilized eggs (lane 1, 100 μg/lane) and 293 human embryonic kidney cells (lane 2, 20 μg/lane) were separated by SDS-PAGE and analyzed by immunoblotting with the anti-p85 subunit of PI 3-kinase antibody. An asterisk indicates the position of the immunoreactive 85-kDa protein (doublets in the egg extracts). Molecular size markers (in kDa) used are also indicated. (B) Low density, detergent-insoluble membrane (LD-DIM) (left panels) and detergent-soluble (right panels) fractions were prepared from *Xenopus *eggs that had been untreated (lanes 1 and 6) or activated by 10^6^/ml sperm (lanes 2 and 7), 0.5 μM A23187 (lanes 3 and 8), 10 mM H2O2 (lanes 4 and 9), or 5 U/ml cathepsin B (lanes 5 and 10). Protein samples (LD-DIM fractions, 5 μg/lane; detergent-soluble fractions, 800 μg/lane) were immunoprecipitated with the anti-p85 subunit of PI 3-kinase antibody and the immunoprecipitates were analyzed by immunoblotting with the same antibody (IB: p85) or with the anti-phosphotyrosine antibody (IB: phospho-p85). Asterisks indicate the positions of the p85 bands in each panel. (C) LD-DIM fractions were prepared from *Xenopus *eggs that had been inseminated for the periods indicated (0-40 min). Protein samples (5 μg/lane) were analyzed for the presence of PLCγ, p85 subunit of PI 3-kinase, or tyrosine-phosphorylated UPIII (pUPIII) by immunoprecipitation and immunoblotting (IB) as described in "Methods". Triton X-100-solubilized extracts of *Xenopus *unfertilized eggs (100 μg/lane) were also analyzed for the presence of phosphorylated MAPK (pMAPK) by direct immunoblotting as in Figure 1C. Asterisks indicate the positions of the protein bands of interest.

Immunoblotting demonstrated that an 85-kDa subunit of PI 3-kinase, hereafter termed p85; is expressed in unfertilized *Xenopus *eggs (Figure 2A). Subcellular fractionation experiments demonstrated that p85 is predominantly present in the detergent-soluble fractions of unfertilized eggs (Figure 2B). However, fertilization promoted a transient accumulation of p85 in low-density, detergent-insoluble membranes (LD-DIM or membrane microdomains) (Figure 2C, IB: p85). The sperm-induced translocation of p85 to membrane microdomains (2 min) occurred as early as the transient translocation of PLCγ (Figure 2C, IB: PLCγ, 2 min) and preceded the tyrosine phosphorylation of uroplakin III (Figure 2C, IB: pUPIII, 5 min), both of which are biochemical signs of the activation of Src in fertilized *Xenopus *eggs. These translocation and phosphorylation events were shown to cease in 40-min inseminated egg samples, where dephosphorylation of MAPK is taking place (Figure 2C, IB: pMAPK, 40 min). Translocation of p85 to the egg membrane microdomains was also seen when eggs were treated with either hydrogen peroxide (H2O2) or cathepsin B, which are artificial egg activators involved in the activation of Src (Figure 2B, IB: p85, lanes 4 and 5). On the other hand, translocation of p85 did not occur when eggs were activated by the Ca^2+ ^ionophore A23187 (Figure 2B, IB: p85, lane 3). Under the same experimental conditions, we evaluated the tyrosine phosphorylation of p85 by immunoblotting with anti-phosphotyrosine antibody. It was shown that only the H2O2 treatment of eggs promoted an increase in the tyrosine phosphorylation of p85 (Figure 2B, IB: phospho-p85, lanes 4 and 9). These results suggest that any sperm-induced activation of PI 3-kinase involves the transient translocation of p85 to membrane microdomains, but not increased tyrosine phosphorylation of p85.

**Figure 3:**
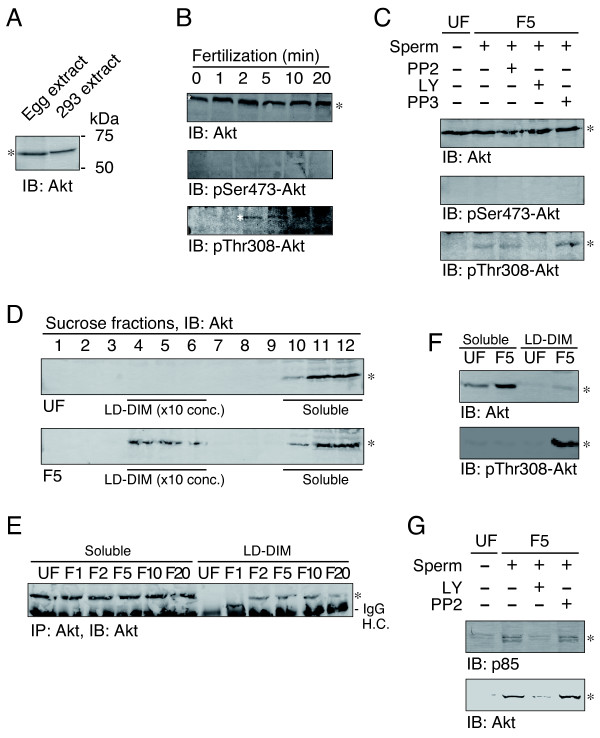
**Phosphorylation and translocation of a serine/threonine-specific protein kinase Akt in fertilized *Xenopus *eggs**. (A) Triton X-100-solubilized extracts of *Xenopus *unfertilized eggs (lane 1, 100 μg/lane) and 293 human embryonic kidney cells (lane 2, 20 μg/lane) were separated by SDS-PAGE and analyzed by immunoblotting with the anti-mammalian Akt antibody. Molecular size markers (in kDa) used are also indicated. (B) *Xenopus *unfertilized eggs were microinjected with DMSO alone, 10 μM PP2, or 10 μM LY294002, and subjected to no treatment (0 min) or insemination for the periods indicated (1-20 min). Triton X-100-solubilized extracts (100 μg/lane) were prepared and analyzed by immunoblotting with the anti-Akt antibody (IB: Akt), anti-phosphorylated serine-473-specific antibody (IB: pSer473-Akt), or anti-phosphorylated threonine-308-specific antibody (IB: pThr308-Akt). (C) Effect of microinjection of PP2 (lane 3), LY294002 (lane 4), or PP3 (lane 5) on sperm-induced phosphorylation of Akt was evaluated using unfertilized (UF, lane 1) and 5-min inseminated egg samples (F5, lanes 2-5) by immunoblotting as in panel B. (D) *Xenopus *unfertilized eggs (UF) and 5-min inseminated eggs (F5) were subjected to subcellular fractionation by discontinuous sucrose density gradient ultracentrifugation as described in "Methods". The twelve fractions obtained were analyzed by immunoblotting with anti-Akt antibody (IB: Akt). The positions of LD-DIM fractions (fractions 4-6, 10-time concentrated) and detergent-soluble, non-microdomain fractions (fractions 10-12) are indicated. (E) LD-DIM fractions and soluble, non-microdomain fractions were prepared from unfertilized eggs (UF) and fertilized eggs (1-20 min after insemination, F1-F20). Protein samples of the same egg-equivalent amounts (about 10 eggs per lane) were analyzed for the presence of Akt by immunoprecipitation and immunoblotting with the anti-Akt antibody (IP: Akt, IB: Akt). (F) Detergent-soluble, non-microdomain fractions (20 eggs per lane) and LD-DIM fractions (2 eggs per lane) that had been prepared from unfertilized eggs (UF) and 5-min inseminated eggs (F5) were analyzed by immunoblotting with either the anti-Akt antibody (IB: Akt) or anti-phosphorylated threonine-308-specific antibody (IB: pThr308-Akt). (G) Effect of LY or PP2 on the sperm-induced translocation of the p85 subunit of PI 3-kinase (upper panel) and Akt (lower panel) was evaluated by immunoblotting of LD-DIM fractions that had been prepared from unfertilized eggs (UF) or 5-min fertilized eggs (F5). In all panels, asterisks indicate the positions of Akt or p85.

We next examined whether the activity of PI 3-kinase is upregulated in fertilized *Xenopus *eggs. To this end, we evaluated the phosphorylation status of Akt, a serine/threonine-specific protein kinase known to be a downstream target of the activated PI 3-kinase. Immunoblotting demonstrated that a 60-kDa Akt protein is present in unfertilized *Xenopus *eggs (Figure 3A, IB: Akt). Fertilization promoted phosphorylation of Akt at threonine-308, one of the activating phosphorylation sites in Akt (Figure 3B, IB: pThr308-Akt). On the other hand, serine-473, another activating phosphorylation site of Akt, remained unphosphorylated in both unfertilized and fertilized eggs (Figure 3B, IB: pSer473-Akt). LY294002, but not PP2, blocked the threonine phosphorylation of Akt (Figure 3C, IB: pThr308-Akt). These results suggest that sperm-induced phosphorylation of Akt at threonine-308 requires the activity of PI 3-kinase, but not the activity of Src tyrosine kinase.

Subcellular fractionation experiments demonstrated that Akt protein undergoes translocation to membrane microdomains in fertilized eggs (Figure 3D). The translocation of Akt occurred as early as 2 min after insemination (Figure 3E), a time course that is similar to translocation of p85 (Figure 2B). Quantitative analysis demonstrated that only a small fraction of Akt localized to membrane microdomains in fertilized eggs (Figure 3F, IB: Akt). However, the threonine-308-phosphorylated forms of Akt were predominantly present in the membrane microdomains (Figure 3F, IB: pThr308-Akt). These results suggest that the sperm-induced functional interaction, and activation, of PI 3-kinase and Akt take place specifically in the egg membrane microdomains. We also examined whether the sperm-induced re-localization of Akt requires the activity of PI 3-kinase. LY294002 was shown to inhibit the sperm-induced appearance of both p85 and Akt in the membrane microdomains (Figure 3G). Like in the case of the threonine-308-phosphorylation described above, PP2 did not affect the re-localization of p85 or Akt (Figure 3G).

**Figure 4:**
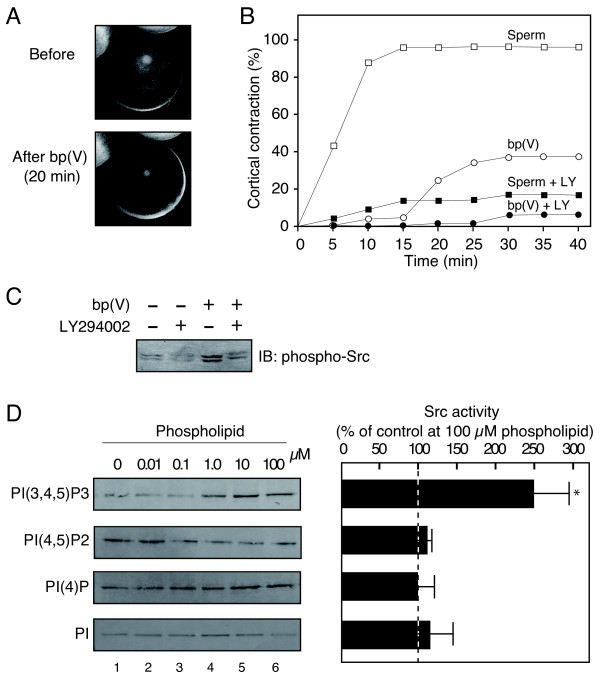
**Activation of *Xenopus *unfertilized eggs by bp(V), a potent inhibitor of PTEN**. (A) Shown are representative photographs of *Xenopus *eggs before and after 20-min treatment with 200 μM bp(V). (B) The occurrence of cortical contraction as a function of time was monitored in *Xenopus *eggs that had been treated with sperm alone (sperm, open squares), 200 μM bp(V) alone (bp(V), open circles), sperm plus pre-injected 10 μM LY294002 (sperm + LY, closed squares), or 200 μM bp(V) plus pre-injected 10 μM LY294002 (bp(V) + LY, closed circles). (C) *Xenopus *eggs that had been treated with or without 200 μM bp(V) and 10 μM LY294002 for 30 min were subjected to extraction of the membrane fractions. The SDS-solubilized membrane extracts (500 μg per lane) were analyzed for tyrosine phosphorylation of Src as in Figure 1A. (D) Purified Src was pretreated with several phospholipids at the indicated concentrations (0-100 μM) and subjected to in vitro kinase assays as described in "Methods". Autophosphorylation of Src was analyzed by immunoblotting of the reaction mixtures with the phosphorylated tyrosine-419-specific antibody. Shown on the left are representative immunoblotting data. Results shown in the right are the mean ± s.e.m. of four independent experiments (at 100 μM of each phospholipids). *P < 0.01 compared with levels in the control. Src activity in the absence of phospholipids was taken as 100%.

To examine further the involvement of the activity of PI 3-kinase in sperm-induced egg activation, we employed bp(V) [dipotassium bisperoxo (picolinato) oxovanadate (V)], a potent inhibitor of the phosphoinositide phosphatase PTEN that converts PIP3, an enzymatic product of PI 3-kinase, into PIP2. As shown in Figure 4A and 4B, bp(V) treatment of unfertilized *Xenopus *eggs induced a cortical contraction, a cytological event of Ca^2+^-dependent egg activation. Importantly, the cortical contraction induced by bp(V) was inhibited by pre-injection of LY294002 (Figure 4B). Moreover, bp(V) treatment of eggs also promoted the tyrosine phosphorylation of Src (Figure 4C). These results demonstrate that artificially elevating the PIP3 level in unfertilized eggs can promote an egg activation-like event as seen in fertilized eggs. Finally, we examined the effect of PIP3 on Src tyrosine kinase activity. An in vitro kinase assay with purified *Xenopus *Src demonstrated that PIP3 was capable of activating the autophosphorylation of Src (Figure 4D). Inositol phospholipids other than PIP3; namely PI, PIP, and PIP2, did not show such a Src-activating property (Figure 4D). These results indicate PIP3 to be a specific and direct activator for Src tyrosine kinase in *Xenopus *eggs.

## Discussion

In *Xenopus *eggs, the sperm-induced activation of Src tyrosine kinase plays an essential role in Ca^2+ ^transient and egg activation [11,43,44]. The present study has indicated the activity of PI 3-kinase to connect egg-sperm interaction/fusion with the activation of Src, because 1) LY294002 inhibits the sperm-induced activation of Src, suggesting that PI 3-kinase acts as an upstream activator for Src, and 2) tyrosine phosphorylation of the p85 subunit of PI 3-kinase, a well-known phenomenon in several cellular systems, in which one or more tyrosine kinase is activated, is not stimulated in fertilized eggs. If so, what is the mechanism for Src activation by PI 3-kinase activity? One possibility is, as demonstrated in the present study, the kinase-activating interaction of PIP3 with Src. The Src homology 2 (SH2) domain of the Src protein is capable of binding PIP3 [45], supporting the possibility that interaction with PIP3 would displace the negative-regulatory intramolecular interaction involving the SH2 domain, leading to the Src kinase's activation.

In our experimental system, Wortmannin, another potent inhibitor for PI 3-kinase, is not inhibitory to the sperm-induced egg activation in *Xenopus*: in eggs injected with Wortmannin at up to 20 μM, sperm could promote Src activation, p85 translocation, cortical contraction, and most importantly Akt phosphorylation, all of which are events of egg activation (data not shown). There is a possibility that, in *Xenopus *eggs, Wortmannin binds preferentially to molecules other than PI 3-kinase. This idea is supported by the finding of Carnero and Lacal [23] that wortmannin is able to induce meiotic maturation in *Xenopus *oocytes at doses slightly higher that those required for complete inhibition of PI 3-kinase. The authors have also suggested that this effect is independent of the ability of Wortmannin to inhibit PI 3-kinase since LY294002 was unable to induce oocyte maturation at concentrations inhibitory to PI 3-kinase, and that the mechanism for wortmannin-induced maturation involves the activation of a maturation-promoting factor and MAPK [23].

A positive regulatory role for PIP3 in egg activation signaling has been suggested by the finding that bp(V), a potent inhibitor for PTEN phosphoinositide phosphatase, promotes tyrosine phosphorylation of Src and cortical contraction of *Xenopus *eggs. (Figure 4A-C) The results indicate that the elevation of PIP3 levels in eggs is sufficient for promoting Src-dependent signal transduction for egg activation.

We demonstrated that, in fertilized eggs, Akt was phosphorylated at Thr308. However, we did not detect the phosphorylation of another well-known site, Ser473. This could be because the phosphoSer473-specific antibody employed in this study was not suitable for analysis of the *Xenopus *Akt protein. However, immunoblotting has demonstrated that the phosphoSer473-specific antibody well recognizes Akt protein in immature *Xenopus *oocytes and that the phosphoSer473 signals become decreased in progesterone-treated, fully matured oocytes (unpublished results). Therefore, we suggest that fertilization promotes the phosphorylation of Akt selectively at threonine-308. In mammals, phosphorylation of Akt Thr308 and Ser473 is catalyzed by phosphoinositide-dependent protein kinase PDK1 [46] and mammalian target of rapamycin mTOR [47], respectively. So, we assume that the signaling pathway involving PDK1, not mTOR, operates to promote the phosphorylation and activation of Akt in fertilized eggs.

Several cellular functions involve the activation of Akt [46,48]. Of particular interest is the anti-apoptotic function of Akt. Our preliminary experiments have shown that a caspase-like protease is activated in eggs left unfertilized for several hours (unpublished results). The results suggest that fertilization is responsible for suppression of the caspase's activation. Therefore, our future efforts will be directed at examining whether an anti-apoptotic mechanism is really operating in fertilized eggs and, if so, to clarify how and where Akt is involved in this cellular function.

Several studies have focused on the roles of sperm-associated PI 3-kinase in mammalian species. In human sperm, LY294002 blocks the acrosome reaction [49] and enhances motility [50], but does not affect egg-sperm interaction [51]. More recently, Jungnickel et al. [52] demonstrated that in the mouse, the zona pellucida glycoprotein 3-induced acrosome reaction involves the PIP3-dependent activation of Akt. These results suggest that PI 3-kinase is required for Ca^2+ ^signaling in fertilizing sperm. In *Xenopus*, however, no information has yet been available for the role of PI 3-kinase and/or Akt in the acrosome reaction or other fertilization-related functions of sperm.

Another important question is how PI 3-kinase could be activated after egg-sperm interaction/fusion (without the involvement of tyrosine phosphorylation)? In this connection, the fact that fertilization is accompanied by a transient accumulation of the p85 subunit of PI 3-kinase and Thr308-phosphorylated form of Akt in the egg membrane microdomains may be important. As we reported earlier, egg membrane microdomains are abundant in cholesterol, the ganglioside GM1, and several signaling molecules, and serve as a platform for egg-sperm interaction and subsequent Src-dependent signal transduction [42,53]. More recently, we have identified and characterized a membrane microdomain-associated protein, uroplakin III, which might act as a target of sperm-derived protease and as a substrate of the activated Src at fertilization [16,54,55]. Although the signal transduction pathway connecting uroplakin III and Src is not yet known, the involvement of GTP-binding proteins has been inferred because the application of GTP to the isolated egg membrane microdomains could reconstitute the Src activity in vitro (Iwasaki et al. manuscript in preparation; 53). So, it is tempting to speculate that PI 3-kinase acts as a downstream target of GTP-binding proteins, as demonstrated in the case of γ-subspecies of PI 3-kinase [56], so that GTP- and PIP3-dependent activation of Src is possible.

The specific activation of PI 3-kinase, however, is not necessarily required for egg activation signaling. Rather, the rapid and transient accumulations of PI 3-kinase at membrane microdomains by itself maybe important. Such a local concentration of PI 3-kinase may contribute to the effective propagation of PIP3-dependent and LY294002-sensitive signals for egg activation. In support of this, PIP2 is known to predominantly localize to membrane microdomains of mammalian cells [57,58]. Further study should be directed toward analyzing the mechanism by which membrane microdomains allow the precise localization of these signaling molecules before and after fertilization.

## Conclusions

In this study, we show that LY294002 effectively inhibited several early steps of sperm-induced egg activation, including the activation of Src. Although we should keep in mind that the effect of LY294002 could be mediated through other targets than PI 3-kinase, we think that the inhibitory effect of LY294002 is most likely due to the inhibition of PI 3-kinase: because not only several steps of Src-dependent egg activation but also sperm-induced phosphorylation of Akt are inhibited by this inhibitor. PI 3-kinase and Akt become activated and temporarily localize to the egg membrane microdomains where sperm-induced tyrosine kinase machinery operates for successful fertilization. These results highlight for the first time the important role the egg-associated PI 3-kinase plays in signal transduction for *Xenopus *fertilization.

## Methods

### Animals, antibodies, cultured cells and chemicals

Frogs were purchased from local dealers and maintained as described [11,42]. Rabbit antibody to human Src phosphorylated at Tyr418 was obtained from Oncogene Research Products (CA, USA). Mouse anti-mouse PLCγ antibody was from Upstate Biotechnology (NY, USA). Rabbit antibodies against the recombinant *Xenopus *UPIII extracellular domain (xUPIII-ED) or a part of the *Xenopus *Src amino-terminal region were prepared as described previously [54,59]. The mouse anti-phosphotyrosine antibody PY99 was purchased from Santa Cruz (CA, USA). Rabbit antibodies against phosphorylated human MAPK, human cyclin B2, and *Xenopus *Mos were from BioLabs (MA, USA) or Santa Cruz Biotechnology. Rabbit antibody against the 85-kDa subunit of human PI 3-kinase was obtained from Upstate Cell Signaling Solution (VA, USA). Rabbit antibodies against human Akt, and the phosphorylated Thr308 or phosphorylated Ser473 form of human Akt were obtained from Cell Signaling Technology (MA, USA). Human embryonic kidney 293 cells were grown in Dulbecco's Modified Eagle's Medium supplemented with 10% fetal calf serum at 37°C/5% CO2 in a humidified incubator. A Src-specific inhibitor, PP2, its inactive analog, PP3, a PLC-specific inhibitor, U73122, and a PI 3-kinase-specific inhibitor, LY294002, were purchased from Calbiochem. A23187 and bovine spleen cathepsin B were from Sigma (MO, USA). Leupeptin was from Peptide Institute (Osaka, Japan). H2O2 was from Santoku Chemical Industries (Tokyo, Japan). (*p*-amidinophenyl)methanesulfonyl fluoride hydrochloride (APMSF) was from Calbiochem (CA, USA). A synthetic tyrosine kinase substrate peptide (Cdk1 peptide) was synthesized and purified as described previously [60]. The fluorescent Ca^2+ ^indicator fura-2 was obtained from Calbiochem. *Xenopus *Src was partially (one column chromatography-processed) or fully (four column chromatography-processed) purified from immature oocytes as described previously [60], and used for kinase assays *in vitro *(see below). [γ-^32^P]ATP was obtained from ICN (OH, USA). A potent PTEN (phosphatase and tensin homolog deleted from chromosome 10) inhibitor bp(V) [dipotassium bisperoxo (picolinato) oxovanadate (V)] was purchased from Calbiochem. Phosphatidylinositol (PI) was obtained from Serdary Research Laboratories (Ontario, Canada). PI 4-phosphate and PI 4,5-bisphosphate were prepared from bovine brain as described previously [61]. PI 3,4,5-trisphosphate (fatty acid moieties are palmitate, C16:0) was prepared as described [62]. All the phospholipids were lyophilized from the chloroform solution and suspended in distilled water or 20 mM Tris-HCl (pH 7.5) by sonication. Protein A-Sepharose was obtained from GE Healthcare Biosciences (Uppsala, Sweden). Unless otherwise indicated, other chemicals were purchased from Sigma, Wakenyaku (Kyoto, Japan), Wako (Osaka, Japan), or Nacalai Tesque (Kyoto, Japan).

### Eggs, sperm, and embryos

The collection of unfertilized eggs and sperm, removal of the jelly layer from eggs, and jelly water treatment of sperm were carried out as described previously [11,42]. The activation of jelly layer-free eggs was done by insemination with jelly water-treated sperm (10^6 ^sperm/ml) or by parthenogenetic activation with the calcium ionophore A23187 (0.5 μM), H2O2 (10 mM), cathepsin B (5 U/ml), or bp(V) (200 μM) for the periods specified in the text. After the activation treatments, egg samples were washed with ice-cold DeBoer's solution containing 110 mM NaCl, 1.3 mM KCl, and 0.44 mM CaCl2, pH 7.2, immediately frozen in liquid nitrogen, and kept at -80°C. Activation was monitored by the occurrence of cortical contraction and first cell cleavage in a group of control eggs.

### Microinjection

To determine the requirement of egg PI 3-kinase activity for *Xenopus *egg activation, the unfertilized eggs were microinjected with LY294002 as described previously [11]. The microinjection of PP2, PP3, U73122, or DMSO alone was also performed. Briefly, stock solutions (5 mM in DMSO) for each compound were diluted with buffer containing 20 mM sodium phosphate, pH 7.4, and 100 mM NaCl. The inhibitor solutions were then loaded into an oil-filled glass capillary (Drummond), with a tip diameter of 10-30 μm, which was connected to a pulse-directed injector system (Nanoject; Drummond). Before being microinjected, dejellied eggs were washed and incubated with injection buffer containing 110 mM NaCl, 1.3 mM KCl, 20 mM MgSO4, 0.1 mM EGTA, and 10 mM chlorobutanol. A quantitative injection of the inhibitor solution (25-50 nl per egg) was conducted under microscopic observation, healed, and the eggs subjected to activation treatment (see above).

### Biochemical fractionation

To analyze the presence and/or the phosphorylation status of proteins of interest, egg samples were homogenized or vortex-crushed with 10 μl per egg of ice-cold extraction buffer containing 1% (w/v) Triton X-100, 20 mM Tris-HCl, pH 7.5, 150 mM NaCl, 1 mM EDTA, 1 mM EGTA, 10 mM β-mercaptoethanol, 1 mM Na3VO4, 10 μg/ml leupeptin, and 20 μM APMSF. The extract was centrifuged at 12,000 *g *and the supernatant was used as the Triton X-100-solubilized egg extract (note that the extract contained Triton X-100-resistant membranes). In some experiments, egg samples were subjected to the preparation of crude membrane fractions as described [60]. The membrane fractions were solubilized with the extraction buffer containing 0.1% SDS, and subjected to further experiments. Alternatively, eggs were subjected to the preparation of membrane microdomains as described previously [63]. Briefly, eggs were homogenized with a 5-fold volume of ice-cold extraction buffer without Triton X-100. The homogenates devoid of debris were centrifuged at 150,000 *g *for 1 h. After the centrifugation, the fluffy layer of the pellets, rich in plasma membranes, were collected and adjusted to 1% Triton X-100 (w/v) and 42.5% (w/v) sucrose. The mixtures were then layered with 30% (first) and 5% (second) sucrose in the extraction buffer and centrifuged at 200,000 *g *for 24 h in an SW55 rotor (Beckman). After the centrifugation, aliquots of 12 fractions were collected from the top to bottom of the tubes. Fractions 3-6 were pooled as low density, detergent-insoluble membrane fractions (membrane microdomains) whereas fractions 9-12 were pooled as detergent-soluble, non-microdomain fractions. Protein concentrations were determined by the dye-binding assay (Bio-Rad, CA, USA).

### Immunoprecipitation, immunoblotting, and SDS-PAGE

Protein samples (50-500 μg, 1 mg/ml) were immunoprecipitated with antibodies as specified in the text for 2 h at 4°C. The immune complexes were adsorbed onto 10 μl of protein A-Sepharose beads by gentle mixing for 30 min at 4°C. The beads were washed three times with 500 μl of buffer containing 0.1% SDS, 1% Triton X-100, 1% sodium deoxycholate, 150 mM NaCl, 50 mM Tris-HCl, pH 7.5, 1 mM Na3VO4, 10 μg/ml leupeptin, and 20 μM APMSF. The washed beads were treated with Laemmli's SDS sample buffer [64] at 98°C for 5 min. SDS-denatured proteins were separated by SDS-polyacrylamide gel electrophoresis using 10% gels and analyzed by immunoblotting as described previously [42].

### In vitro kinase assay

The protein kinase assay was carried out in a standard reaction mixture (25 μl) containing an immunoprecipitated or purified *Xenopus *Src, 50 mM Tris-HCl (pH 7.5), 5 mM MgCl2, 2 μM [γ-^32^P]ATP (3.7 kBq/pmol), 1 mM dithiothreitol, and 1 mM Cdk1 peptide. Anti-pepY antibody, which recognized the autophosphorylation site of Src, and SDS-solubilized egg membrane fractions were used for immunoprecipitation as described previously [43,60]. When the effect of several phospholipids was evaluated, purified *Xenopus *Src and non-radioactive ATP (at 1 mM) were employed, and Cdk1 peptide was omitted. The reaction was initiated by the addition of [γ-^32^P]ATP or non-radioactive ATP at 30°C for 10 min and terminated by the addition of SDS-sample buffer [64] followed by boiling for 3 min. Phosphorylation of Cdk1 peptide (radioactive assay) was analyzed by separating the reaction mixtures on 18% SDS-polyacrylamide slab gels, followed by visualization and quantification by a BAS2000 Bioimaging Analyzer (Fuji Film, Tokyo, Japan). Autophosphorylation of *Xenopus *Src (non-radioactive assay) was analyzed by separating the reaction mixtures by using 10% SDS-polyacrylamide slab gels, followed by immunoblotting with the anti-phosphorylated Tyr418 antibody.

### Intracellular Ca^2+ ^measurement

For Ca^2+ ^measurements, dejellied eggs prepared from albino *Xenopus *females were co-microinjected with 50 nl of 160 μM fura-2 plus 200 μM LY294002 (see "*Microinjection*"). The injected eggs were then fertilized and fluorescent signals were recorded by ratio-imaging microscopy using a high-frame digital CCD imaging ARGUS/HISCA system (Hamamatsu Photonics, Japan) with a 10× planneofluar objective, NA 0.3 (Zeiss, Germany). Excitation wavelengths of 340 and 380 nm (each irradiation constant was set to about 800 ms) were used while monitoring the emission at 510 nm. All data collections were made at 10-s intervals at 18-21°C.

## Authors' contributions

MG acquired the experimental data and prepared a preliminary draft. TI, AAT, and YF participated in the design of the study and contributed critical comments and suggestions while performing experiments and preparing drafts. KS organized the research, obtained data, interpreted results, and revised and finalized the manuscript. All authors read and approved the final manuscript.
